# Identification of Visual Attentional Regions of the Temporoparietal Junction in Individual Subjects using a Vivid, Novel Oddball Paradigm

**DOI:** 10.3389/fnhum.2019.00424

**Published:** 2019-12-11

**Authors:** Kathryn J. Devaney, Maya L. Rosen, Emily J. Levin, David C. Somers

**Affiliations:** ^1^Department of Psychological and Brain Sciences, Boston University, Boston, MA, United States; ^2^Department of Health and Human Performance, Stanford University Medical School, Stanford, CA, United States; ^3^Department of Psychology, Harvard University, Seattle, WA, United States; ^4^Department of Cognitive, Linguistic and Psychological Sciences, Brown University, Providence, RI, United States

**Keywords:** attention, temporal lobe, parietal lobe, magnetic resonance imaging, individual-subject approach, TPJ

## Abstract

The Temporoparietal Junction (TPJ) of the cerebral cortex is a functionally heterogeneous region that also exhibits substantial anatomical variability across individuals. As a result, the precise functional organization of TPJ remains controversial. One or more regions within TPJ support visual attention processes, but the “attention TPJ” is difficult to functionally observe in individual subjects, and thus is typically identified by averaging across a large group of subjects. However, group-averaging also blurs localization and can obscure functional organization. Here, we develop and test an individual-subject approach to identifying attentional TPJ. This paradigm employs novel oddball images with a strong visual drive to produce robust TPJ responses in individuals. Vivid, novel oddballs drive responses in two TPJ regions bilaterally, a posterior region centered in posterior Superior Temporal Sulcus (TPJ_STS_) and an anterior region in ventral Supramarginal Gyrus (TPJ_SMG_). Although an attentional reorienting task fails to drive TPJ activation in individuals, group analysis of the attentional reorienting contrast reveals recruitment of right TPJ_STS_, but not right TPJ_SMG_. Similarly, right TPJ_STS_, as identified in individual subjects by the vivid, novel oddball contrast, is activated by attentional reorienting, but right TPJ_SMG_ is not. These findings advance an individual-subject based approach to understanding the functional organization of TPJ.

## Introduction

The human cortical temporoparietal junction (TPJ) has been implicated in a wide range of cognitive processes including attentional reorienting (Corbetta et al., 2000; Kincade et al., 2005; Astafiev et al., 2006; Fox et al., 2006; Shulman et al., 2009; Han and Marois, 2014), contextual updating (DiQuattro and Geng, 2011; Geng and Vossel, 2013; DiQuattro et al., 2014), and social cognition (Young and Saxe, 2008, 2009; Saxe, 2010; but see Mitchell, 2008). The human TPJ also demonstrates a high degree of individual variability both anatomically and in its connectivity profile (Hasson et al., 2004; Van Essen, 2005; Mueller et al., 2013). The TPJ is one of the areas of largest cortical expansion from macaques to humans (Buckner and Krienen, 2013), and thus non-human primate models provide fewer clues to functionality in this region of the human brain. It is likely that TPJ consists of multiple, functionally distinct regions. For instance, a posterior TPJ may serve Theory of Mind functions, while an anterior TPJ serves attention functions (Krall et al., 2015; Trautwein et al., 2016). However, other studies suggest that finer-scale organization, with as many as five distinct regions in a hemisphere, may exist within TPJ (Igelström et al., 2015, 2016; Dugué et al., 2018). In this manuscript, we investigate and localize in individual subjects a set of visually-responsive TPJ sub-regions that are modulated by attention.

The location of the attentional TPJ is highly variable between individuals (Hasson et al., 2004; Van Essen, 2005; Niogi et al., 2010; Mueller et al., 2013; Dugué et al., 2018) and thus between-subject group averaging is not the ideal approach to localizing this region. Although sophisticated data analysis techniques have been applied to group resting-state (Mars et al., 2012b; Igelström et al., 2015) or functional data (Igelström et al., 2016) to produce detailed TPJ subdivisions, these approaches have yielded somewhat inconsistent parcellations of TPJ. Additionally, given the apparent proximity of a Default Mode Network region (which is suppressed by many attentionally demanding tasks) to a region activated by attentional reorienting (e.g., Mars et al., 2012a), analyses that rely on group-averaging techniques to identify a single TPJ could easily be partly confounded by the inclusion of heterogeneous functional domains into the region of interest (ROI). We suggest that careful within-subject mapping across multiple functional tasks (e.g., Somers and Sheremata, 2013; Michalka et al., 2015) could prove useful for yielding a clearer understanding of the functional organization of the TPJ region. A key goal of the present study is to validate an functional magnetic resonance imaging (fMRI) method to reliably localize the visual attentional TPJ region or regions across individual subjects.

In prior work, attention functions in TPJ typically have been localized with one of three broad paradigms. The most commonly employed paradigm is a Posner-style spatial cueing and reorienting task (Posner, 1980; Thiel et al., 2004; Weissman and Prado, 2012; de Haan et al., 2015) in which participants report the appearance of a stimulus at an attended (valid) or unattended (invalid) location; attentional TPJ can be identified by the contrast of invalid trials geater than valid trials because correct performance on invalid trials requires a reorienting of attention. A second approach involves the presentation of infrequent, salient, unexpected “oddball” stimuli; oddball stimuli induce attentional capture and thus the contrast of oddball greater than non-oddball trials can be used to identify attentional TPJ (Sutton et al., 1965; Linden et al., 1999; Clark et al., 2000; Bledowski et al., 2004; Asplund et al., 2010; Kim, 2013; Warbrick et al., 2013). A third, less commonly applied approach is to use direct visual drive to elicit responses in TPJ (Horiguchi et al., 2016; Dugué et al., 2018). This visual drive approach is very promising in that it has been successful in identifying TPJ in individual subjects. Dugué et al. (2018) observed two bilateral and one right hemisphere visually-driven TPJ. Horiguchi et al. (2016) consistently observed only a single right hemisphere TPJ, indicating that their approach may have been underpowered to observe the full set of regions reported by Dugué et al. (2018). We summarize findings from all approaches used to identify TPJ in a meta-analysis, displayed in Table 1.

**Table 1:** Studies included in meta-analysis.

References	Talairach	Task
Astafiev et al. (2006)	51 −51 26	Other
Braver et al. (2001)	58 −48 24	Oddball
Chen et al. (2012)	48 −42 14	Cueing
Corbetta et al. (2000)	53 −49 30	Cueing
Corbetta et al. (2002)	57 −45 12	Cueing
Davis et al. (2009)	54 −41 23	Other
de Haan et al. (2015)	50 −43 21	Cueing
Doricchi et al. (2010)	60 −46 28	Cueing
Downar et al. (2001)	54 −43 17	Other
Downar et al. (2002)	56 −36 24	Oddball
Dugué et al. (2018)	45 −61 9	Other
Geng and Mangun (2011)	42 −57 14	Other
Giessing et al. (2006)	40 −46 18	Cueing
Gillebert et al. (2012)	47 −32 −7	Oddball
Himmelbach et al. (2006)	55 −57 16	Other
Horiguchi et al. (2016)	52 −34 21	Other, Oddball
Indovina and Macaluso (2007)	46 −37 26	Other
Jakobs et al. (2012)	54 −47 25	Rest
Kincade et al. (2005)	50 −48 26	Cueing
Kucyi et al. (2012)	57 −37 27	Other
Lee and McCarthy (2016)	50 −41 23	Cueing
Lepsien and Pollmann (2002)	56 −52 16	Cueing
Luks et al. (2010)	48 −54 7	Other
Macaluso et al. (2002)	60 −48 32	Cueing
Mayer et al. (2004)	54 −51 28	Cueing
Mitchell (2008)	60 −48 27	Cueing
Pagnoni (2012)	58 −46 25	Other
Ptak and Schnider (2011)	45 −53 25	Oddball
Ruff and Driver (2006)	56 −36 16	Cueing
Scholz et al. (2009)	58 −62 42	Cueing
Serences et al. (2005)	55 −44 24	Other
Shulman et al. (2003)	51 −48 28	Other
Shulman et al. (2009)	52 −49 17	Other
Silvetti et al. (2016)	60 −46 28	Cueing
Thiel et al. (2004)	41 −65 15	Cueing
Todd et al. (2005)	59 −47 24	Other
Trautwein et al. (2016)	38 −62 16	Cueing
Tyler et al. (2015)	49 −61 20	Cueing
Uncapher et al. (2011)	58 −38 34	Oddball
Vetter et al. (2011)	37 −54 20	Other
Vossel et al. (2006)	56 −55 17	Cueing
Vossel et al. (2009)	61 −44 11	Cueing
Weissman and Prado (2012)	44 −64 12	Cueing
Woldorff et al. (2004)	58 −45 16	Cueing
Wu et al. (2015)	50 −52 22	Oddball

Here, we combine all three elements, spatial reorienting, oddballs, and visual drive in a single scan task paradigm. Our key manipulation utilizes vivid, novel oddball stimuli that drive both strong visual and strong novelty responses. Our rationale is that by combining both factors we may elicit a robust enough transient fMRI response that it can be reliably detected in individual subjects. We use full-screen vivid images (outdoor scenes) in order to produce a strong visual drive. Due to prior work indicating that repeated presentation of a stimulus weakens the oddball response (Asplund et al., 2010), we present a novel image for every oddball occurrence. We refer to this as a “vivid, novel oddball” (ViNO) paradigm. The inclusion of a spatial cueing task within the same scan runs permits us to compare activation across task components within individual subjects.

## Materials and Methods

### Participants

Ten healthy right-handed individuals (four males), ages 27–33 with normal or corrected-to-normal vision participated. All participants had no history of neuropsychological disorders. All were recruited from Boston University and the greater Boston community and gave written informed consent. All experiments were approved by the Institutional Review Board of Boston University. Participants were compensated monetarily for their time.

### Combined Spatial Cueing and Vivid, Novel Oddball (ViNO) Paradigm

**Figure 1:**
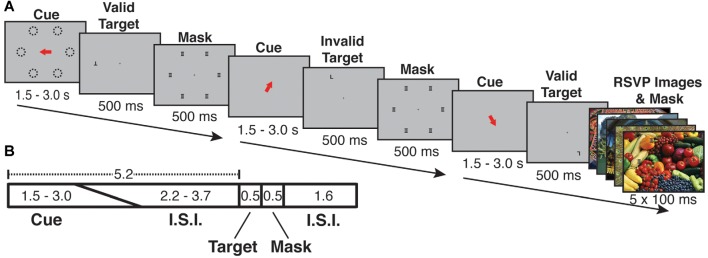
Cue-ball task trial structure and timing. **(A)** Examples of each of three trial types (Valid, Invalid, Oddball). Each trial began with a spatial cue indicating one of six possible locations (dashed lines in **A**, dashed lines were not visible during the experiment). Following an inter-stimulus interval, the target appeared. The target was the letter “T” or “L” and could appear upright or inverted. The cue could be valid (target appears at the cued location) or invalid (appears in an uncued location). Following the target, a mask of superimposed “T”s and “L”s was presented at all possible target locations. On a randomly selected 1/6 of trials, a task-irrelevant RSVP stream of five vivid, full-screen, session-unique “oddball” images appeared behind the letter mask, each with 100 ms duration. **(B)** Trial timing: the orienting phase was 5.2 s in total, with cue duration randomly jittered from 1.5 to 3.0 s and the fixation cross ISI accounting for the remaining 5.2 s. Following the orienting phase, the target appeared for 500 ms, followed by the mask or oddball mask for 500 ms. The mask/oddball phase was followed by an inter-trial interval of 1.6 s. Participants were asked to indicate the identity of the target letter (T or L).

Participants performed an endogenous, covert spatial attention task that was interrupted by infrequent and task-irrelevant oddball events in the form of vivid, novel, full-screen images (Figure 1). The attention task required participants to maintain central fixation and to indicate the identity of a target letter (T or L, presented at 7 degrees eccentricity and at either 0° or 180° of rotation) following a spatial cue period.

Each trial lasted 7.8 s and consisted of the following phases: orienting phase (cue and ISI), target phase (letter on screen), mask phase (mask on screen) and ITI (fixation cross on screen). During the orienting phase, a red arrow cue appeared and indicated one of six possible target locations on the screen. To encourage attention to only the cued location, participants were instructed in advance that the letter target would appear at the spatially cued location “more often than not,” and that the letter target would never appear at a location other than the six possible cued locations. The timing of the orienting phase was jittered such that the duration of the cue and ISI together always summed to 5.2 s, with the cue taking a random value between 1.5 and 3.0 s and the ISI filling the rest of the 5.2 s; the unpredictable interval from cue offset to target onset encouraged the deployment of sustained attention over the full ISI. Following the orienting phase, the target (upright or inverted T or L) appeared for 500 ms either at the cued (valid) or an uncued (invalid) location. Invalid targets always appeared in a random location (one of three) in the uncued hemifield. Spatial cues were valid on 68.75% of trials (33 out of 48 trials per run). Participants responded with one of two keys on a button box to indicate the identity of the letter target regardless of its orientation or location. The target was followed by a 500 ms mask of superimposed T’s and L’s at all of the six possible target locations (Figure 1). Following the mask, participants were shown a fixation cross for the ITI of 1.6 s. Responses were recorded from target onset to the start of the following trial (a 2.6 s total possible response period). Each run consisted of 48 trials, and participants completed either 10 (s05, s06) or 12 (all other subjects) runs per scan session.

In each run, on a fraction of trials, the post-target mask was displayed with a background of five full-screen (approximately 22 × 16.5 degrees), full-color, unique “oddball” images each presented for 100 ms (Figure 1). Prior work indicates that distractor surprise effects rapidly diminish with repeated presentation of the stimulus (Asplund et al., 2010). In order to keep the surprise effects strong across the scan session, each oddball image was displayed only once to any subject. An additional design consideration was the frequency of oddball trials. A greater number of oddball trials could potentially increase statistical power; however, at high frequency oddballs might come to be expected by subjects, thus reducing BOLD activation per presentation. To examine this issue empirically, we performed a pilot study with four participants, in which we varied the frequency of oddball events parametrically from 1/8 to 1/3 of total trials. The comparison of net BOLD signal vs. oddball frequency exhibited an “inverted-U” shape, indicating that the effects were primarily driven by surprise effects rather than by stimulus drive. The most consistent robust effects were exhibited when oddballs occurred on 1/6 of trials (see Supplementary Figure S1). Therefore, in all subsequent sessions, oddballs were presented on a randomly selected 1/6 of trials. Oddball images were presented in a “rapid-fire” or rapid serial visual presentation (RSVP) burst (five images, 100 ms per image). The intention of the “rapid-fire” oddball sequence was to produce a strong, unexpected attentional capture effect. During 12 runs, participants saw 576 total trials including 96 oddball trials (480 unique oddball images). A diverse variety of vivid and lively scenes that serve as strong exogenous cues to attention were selected for the oddball images. Because prior work (Engell and Haxby, 2007; Tsao and Livingstone, 2008; Nasr et al., 2014) indicates the presence of a visually responsive face-processing region in posterior superior temporal sulcus (STS), images with prominent human faces were excluded from the oddball image set.

Each run also included 7.8 s of fixation (no stimuli other than a fixation cross) at both the start and the end of the run. Each participant was scanned on either 480 or 576 total trials, including 80 or 96 total oddball trials, plus one practice run outside of the scanner. The practice runs did not contain any oddball images and participants were not informed that oddball images would occur, only that they should indicate the identity of the target letter despite variations in its location or orientation, or any other stimuli that might occur. Task paradigm code is available upon request (somers@bu.edu, kdevaney@stanford.edu).

### Test-Retest

Two participants were scanned on two pilot versions of the task [one in which oddball frequency was varied parametrically, as described in “Materials and Methods” section, and one with oddball stimuli occurring in only four runs (time point 1 in Figure 4)]. The low-level stimulus features of the oddball images differed over these three sessions: for each session, the participants saw a random selection of images from a pool of 500 total images.

**Figure 2:**
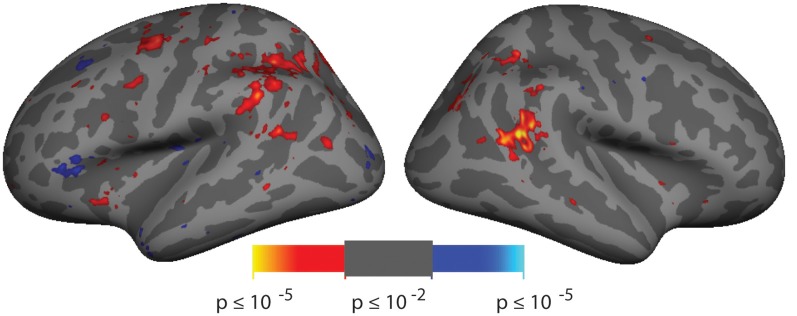
Spatial cueing group-averaged activation. A group-average analysis (*N* = 10) of invalidly (hot colors) vs. validly (cool colors) cued targets (*p* < 0.01, uncorrected). Activation is observed in right hemisphere around the posterior end of the superior temporal sulcus (STS) near the border with the parietal lobe.

**Figure 3:**
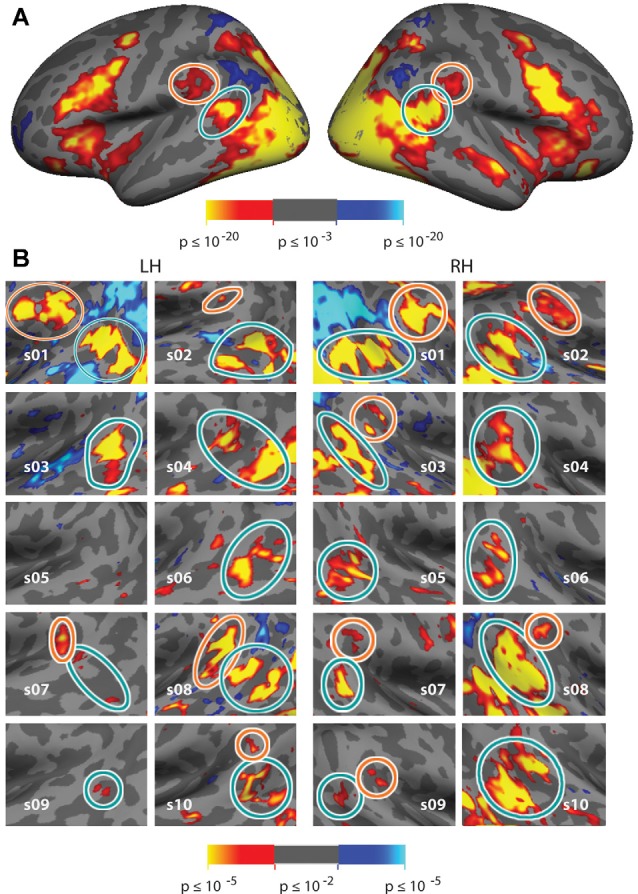
Vivid, Novel Oddball effect in group average and in individual subjects. **(A)** Group average analysis (*N* = 10; cluster correction threshold of *p* < 0.01) of oddball vs. non-oddball trials reveals robust activation in bilateral temporoparietal junction (TPJ) and indicates two distinct patches of activation falling within the region of the TPJ: a large bilateral posterior patch (“TPJ_STS_,” cyan circle), spanning both banks of the STS, and a smaller bilateral anterior patch (“TPJ_SMG_,” orange circle) in the ventral portion of the Supramarginal Gyrus (SMG). **(B)** Zoom-in of the same analysis shown in each individual subject (*p* < 0.01, uncorrected; see also Supplementary Figure S4 for contrast effect sizes).

**Figure 4:**
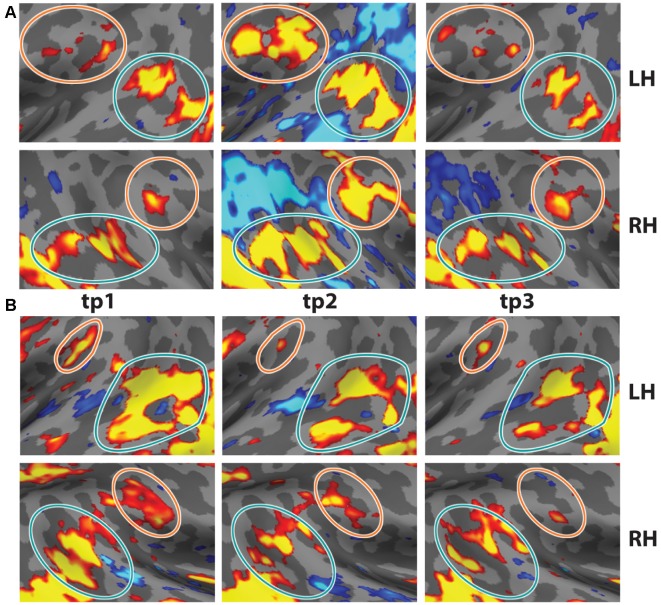
Test-Retest of oddball contrast in two individual subjects. Two subjects were scanned on the task three times. **(A)** Data from s01 at *t* = 0, *t* = 7 months, and *t* = 19 months. **(B)** Data from s02 at *t* = 0, *t* = 5 months, and *t* = 11 months (*p* < 0.01, uncorrected). Note the consistency across time in the anatomical locations. Data from tp1 reflects four runs of the task.

### MR Imaging Procedures and Data Analysis

#### MR Scanning

All task data were acquired using a 32-channel Siemens head coil in a horizontal bore 3 Tesla Siemens Tim Trio located at the Harvard University Center for Brain Science in Cambridge, MA, USA. Gradient echo EPI sequences were used for all tasks (TR = 2,600 ms, TE = 30 ms, Flip angle = 90°, voxel size = 3.0 × 3.0 × 3.1 mm, 42 slices, whole-brain coverage). Eight participants completed 12 runs (1,728 volumes) and two participants completed 10 runs (1,440 volumes). Magnetization Prepared Rapid Gradient Echo (MP-RaGE) T1-weighted high-resolution data (TR = 6.6 ms, TE = 2.9 ms, Flip angle = 8°, voxel size = 1.0 × 1.0 × 1.3 mm) was acquired for each participant. For nine participants these data were acquired on the same scanner as the task data and for one participant, it was acquired with identical hardware at the Martinos Center for Biomedical Imaging in Charlestown, MA, USA.

#### Analysis

Cortical reconstruction and volumetric segmentation of the T1 data was performed with the Freesurfer image analysis suite, which is documented and freely available for download at http://surfer.nmr.mgh.harvard.edu/. The technical details of these procedures are described in prior publications (Dale and Sereno, 1993; Dale et al., 1999; Fischl et al., 1999a,b; Fischl et al., 2001, 2002; Fischl et al., 2004; Fischl and Dale, 2000; Fischl, 2004; Ségonne et al., 2004; Han et al., 2006; Jovicich et al., 2006). All task data were analyzed with fs-fast version 5.1.0 and custom Matlab scripts. For the task data, motion correction, intensity normalization and boundary-based registration (Greve and Fischl, 2009) to the participant’s own anatomical data were performed on a per-run basis. Singular value decomposition reduced the six motion correction vectors to three eigenvectors, which were included as nuisance regressors in the model. Individual subject analysis concatenated data from all runs into a single time-course, using standard FreeSurfer FS-Fast V5.1 methods. Analyses were performed separately in each hemisphere on each subject’s own cortical surface with 3 mm fwhm smoothing and data were analyzed for each voxel using a GLM with each condition as a predictor (i.e., one for Valid Target, Invalid Target, Oddball, Non-Oddball and Passive). Since invalidly cued oddball trials were necessarily rare (5.2% of trials), interaction effects were not examined. The BOLD signal was modeled as a linear, time-invariant system with a γ response function assumed for each condition with a delay *δ* = 2.25 and a delay time constant *τ* = 1.25. An estimated response was generated by convolving the response function with the event length (i.e., the time in each condition) and minimizing the residual error (FS-FAST). Event-related GLM analysis was performed for each voxel to obtain a beta weight for each condition. Contrast effect sizes were computed for each voxel for each contrast by comparing the beta values. t-stats for the contrasts between conditions were computed using the contrast effect sizes for the conditions and their variance. Activation differences were visualized on the surface of each subject’s own hemisphere using *p*-values derived from these t-stats and applying a liberal threshold of *p* < 0.01 (uncorrected) at the vertex level. Individual subject analysis was restricted to the anatomical vicinity of TPJ, where significant group-level activation was observed (see Figure 3B). Observed clusters of these activated cortical vertices on these maps were used to define individual subject TPJ ROIs that are used in some analyses. The Posner cueing analysis was conducted with the contrast of Invalid vs. Valid Targets and was compared with regions activated for the ViNO contrast of Oddball trials vs. Non-Oddball trials.

For the group average analysis, each participant’s fMRI data were registered to an average cortical surface space (Freesurfer 5.1.0, “fsaverage” brain) using the boundary of the gray matter and white matter (Greve and Fischl, 2009). The GLM analysis methods were the same as for individual subject data, however, the significance of these activation differences was computed on vertices of the fsaverage brain and visualized on that surface. We then employed a random-effects model to compute the group-averaged value for each condition at each vertex before running *t*-tests at each vertex to compare group-level activation differences per condition. Significant group-level task activation was corrected for multiple comparisons using cluster-based correction, permuting the sign on the design matrix (using the FS-FAST tool mri_glmfit-sim) with cluster-thresholding at a corrected alpha of *p* < 0.01. This analysis generated a mask of significant clusters and group-level maps reflect the voxelwise *p-values* within the significant clusters.

## Results

### Meta-Analysis

We reviewed the literature on fMRI studies of the TPJ, focusing on investigations of visual attention, oddball processing, and related topics. The primary type of task contrast used and the Talairach coordinate of peak activation in the right hemisphere TPJ for each study is summarized in Table 1 (see also Geng and Vossel, 2013; Dugué et al., 2018). Across all studies, the mean Talairach position was 52.1 ± 1.0, −48.4 ± 1.2, 20.9 ± 1.2 (*std. err. of mean*). Examination of only the spatial cueing tasks yielded a similar mean Talairach position of 52.7 ± 1.5, −50 ± 1.7, 21.3 ± 1.7. Examination of the oddball contrasts yielded a mean Talairach position of 52.3 ± 2.3, −43.2 ± 3.7, 20.3 ± 5.7, which suggests that the activation for oddball stimuli may shift somewhat anterior relative to the activation due to spatial cueing. However, even within the set of spatial cueing studies, the range of reported Talairach coordinates was very large, 38 ≤ × x 61, −65 ≤ y ≤ −36, 11 ≤ z ≤ 42, and encompassed the mean coordinates for the oddball activation.

### Behavior

Target discrimination (T vs. L) performance was analyzed for three different classes of trials: invalidly cued targets, validly cued targets, and targets followed by vivid, novel oddball stimuli. Median reaction times for each condition in individual subjects were entered into paired *t*-tests across conditions (non-oddball valid vs. invalid, oddball valid vs. invalid). Mean overall accuracy (across all trials and all subjects) was 86 ± 1.6%. There was a trend toward a speed-accuracy tradeoff in this dataset (speed-accuracy correlation *r* = 0.57, *p* = 0.08), therefore only correct trials were included in the RT analysis. As expected, reaction time on non-oddball invalidly cued trials was significantly longer than non-oddball validly cued trials (mean invalid = 724 ± 19 ms, mean valid = 683 ± 18 ms, mean difference = 40 ± 6 ms, *t*_(9)_ = −3.54, *p* < 0.01). Mean reaction time on oddball image trials was more variable (mean = 679 ± 40 ms) and was not significantly different from either type of non-oddball trial.

### fMRI Analysis

Analysis of the spatial cueing conditions contrasted invalidly cued and validly cued conditions. Group-average comparison of invalid to valid cue trials (*n* = 10; *p* < 0.01, uncorrected) revealed activation in the vicinity of the TPJ. The most robust activation was observed in right hemisphere (rh), spanning both banks of a posterior segment of the STS, near the border with the parietal lobe (see Figure 2). With this liberal threshold, weaker activation can be observed in the left hemisphere in a symmetric region in posterior STS and in Supramarginal Gyrus (SMG). Modest group-level activation is also observed in parietal and frontal regions of the dorsal attention network. When analyzed with cluster-correction methods (*p* < 0.01), only the rh STS region survives (see Supplementary Figure S2A). Activation maps for individual subjects were typically blank brain images (see Supplementary Figure S2B). The pattern and amplitude of the activations observed here are similar to prior fMRI studies of spatial cueing (see Table 1). We note that the weak activation in the invalid vs. valid contrast and the inability to observe activation for this contrast in individual subjects was expected based on prior studies and was a primary motivator of our effort to develop a more robust TPJ localizer.

Comparison of trials on which oddball images appeared to non-oddball trials yields much stronger activation than does the spatial cueing contrast. This “ViNO contrast” of vivid, novel oddball trials to non-oddball trials localizes TPJ structures at the group-average level and in individual subjects. Figure 3A shows the group average task-evoked activity maps for the oddball trials compared to non-oddball trials (*n* = 10, random effects model; cluster-corrected *p* < 0.01; see Supplementary Figure S3 for medial surface). The visual attentional TPJ, localized with vivid, novel oddball stimuli, is divided into a posterior region centered on the fundus of and spanning both banks of the STS, that we refer to as TPJ_STS_ (see cyan outline) and an anterior region in the ventral portion of SMG, that we refer to as TPJ_SMG_ (see orange outline). These regions are observed bilaterally. Activation was also observed in regions of frontal, occipital, and dorsal parietal lobe, but analysis of these regions is beyond the scope of this manuscript.

In order to better reveal the visual attentional functional organization around TPJ, we examined activation in the oddball vs. non-oddball contrast in each of 10 individual subjects (see Figure 3B); this analysis was anatomically restricted to the anatomical vicinity of the TPJ activation observed in the group analysis. In order to define TPJ ROIs individual subject hemispheres, these maps were liberally thresholded at *p* < 0.01, uncorrected (see Supplementary Figure S4 for contrast effect sizes). Note that equivalent analysis of individual subjects in spatial cueing trials (invalid vs. valid) yielded very few activated vertices (see Supplementary Figure S2B). The oddball-responsive TPJ in individual subjects consists of posterior and anterior regions with variable anatomical location and magnitude of activation. The posterior TPJ (TPJ_STS_) lies toward the dorsal end of the STS (with a caudal border with occipital areas defined by the caudal aspect of STS). Most commonly, activation spans opposing banks of a small segment of STS. The anterior TPJ (TPJ_SMG_) lies in the ventral portion of the SMG. All subjects (10/10) display a right posterior TPJ (TPJ_STS_) and 9/10 subjects display a left TPJ_STS_. 6/10 subjects display a right anterior TPJ (TPJ_SMG_) and 5/10 subjects display a left TPJ_SMG_ (Table 2). In many subjects, TPJ_STS_ activation splits into two distinct regions, one on each bank of STS; however, this could reflect a partial-volume effect as the two STS banks are often in such close proximity that our voxel resolution does not clearly distinguish them. On the other hand, neither bank of STS is consistently more strongly or extensively activated, as would be expected in partial-voluming and in some subjects a single contiguous pattern of activation is observed to span both banks STS.

**Table 2:** Vivid, Novel Oddball-localized TPJ areas in individual subjects.

				Tal		
ROI	*N* (Hemispheres)	Surface area (mm^2^)	Std. Err.	*X*	*Y*	*Z*
rh TPJ_SMG_	6/10	356	±118	51.44	−30.27	32.29
lh TPJ_SMG_	5/10	394	±152	−48.39	−37.58	27.87
rh TPJ_STS_	10/10	847	±138	44.05	−43.58	16.48
lh TPJ_STS_	9/10	659	±140	−47.47	−47.32	17.61

### Test–Retest

In order to demonstrate the reliability and reproducibility of the TPJ results observed with vivid, novel oddballs, we performed a test-retest analysis with two participants, scanned in three separate sessions each. Subject 01 was scanned 5 months and 11 months after the first session. Subject 02 was scanned 7 months and 19 months after the first session. Test-retest results are shown in Figures 4A,B. The bilateral activity in TPJ_STS_ and TPJ_SMG_ is stable over long time intervals. Note the small but stable patch of activation in the LH TPJ_SMG_. There were minor differences in the oddball paradigm across sessions (see “Materials and Methods” section), but the regions were robustly identified in each scan. This is an important validation of the utility of the ViNO paradigm for TPJ identification in individual subjects.

### Comparison of Spatial Cueing and Vivid, Novel Oddball Activation in TPJ

**Figure 5:**
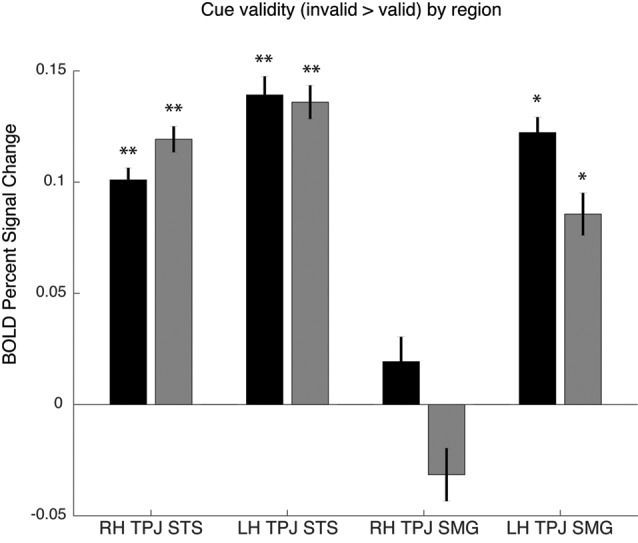
Cue validity effects in oddball-defined regions of interest (ROIs). Individual subject ROIs (black), group-defined ROIs (gray). Percent signal change for invalid vs. valid contrast, *p > 0.05 or **p > 0.01.

As noted above, the spatial cueing contrast failed to reveal substantial TPJ activation clusters in individual subjects, but group-level analysis did reveal activation. In order to examine the Spatial Cueing task contrast activation at a finer scale, we employed the vivid, novel oddball contrast as an individual subject TPJ localizer to identify rh TPJ_STS_, rh TPJ_SMG_, lh TPJ_STS_, lh TPJ_SMG_ and conduct ROI analyses. We examined cue validity effects in individual-subject defined oddball responsive TPJ, in the individuals for whom these ROIs could be defined (see Figure 5). Cue validity effects were evident in bilateral TPJ_STS_ (rh: *n* = 10, *t*_(9)_ = 5.89, *p* = 0.0002; lh: *n* = 9, *t*_(8)_ = 5.57, *p* = 0.0005) and in TPJ_SMG_ in the lh (*n* = 5; *t*_(4)_ = 7.88, *p* = 0.0014), but not the rh (*n* = 6, *t*_(5)_ = 0.71, *p* = 0.5094). The three significant ROIs survive Holm-Bonferroni correction (*p* < 0.01). Since not all individual subjects had all ROIs defined, we repeated this analysis using group-defined ROIs applied to all subjects and obtained very similar results (Figure 5). The invalid vs. valid contrast was significant in the TPJ_STS_ in both the right hemisphere (*t*_(9)_ = 5.77, *p* = 0.0003) and the left hemisphere (*t*_(9)_ = 6.13, *p* = 0.0002). In the TPJ_SMG_, the invalid vs. valid contrast was significant in the left (*t*_(9)_ = 4.93, *p* = 0.0008) but not the right hemisphere (*t*_(9)_ = 1.68, *p* = 0.1273, trending negative). Again, the same three significant ROIs survive Holm-Bonferroni correction (*p* < 0.01). The individual-subject and group-level ROI analyses of these data yield the same conclusions. These results demonstrate that the ViNO contrast is effective in defining TPJ ROIs that capture cue invalidity reorienting effects in individual subjects. Notably, rh TPJ_STS_ was identified in all subjects in this study. This also indicates that rh TPJ_STS_, but not rh TPJ_SMG_ exhibits the functional properties of the TPJ identified in spatial cueing studies.

**Figure 6:**
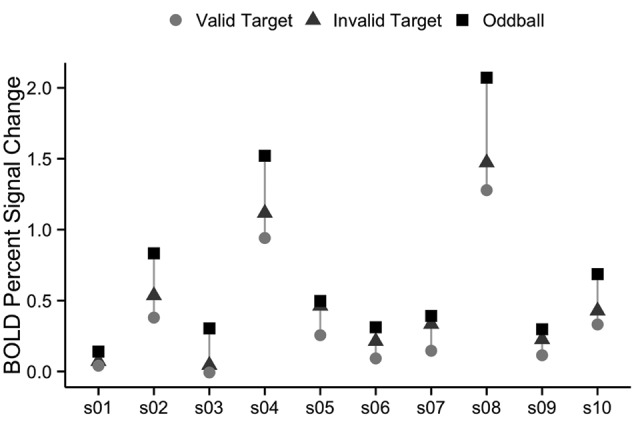
Percent signal change in right TPJ_STS_ for each individual subject, using group-defined ROI from the cueing task. The right TPJ_STS_ ROI was defined using the group average contrast of invalid vs. valid, as shown, in Figure 2. In every subject, oddball stimuli drive right TPJ_STS_ more than invalid cues, even though invalid vs. valid cues were used to define the ROI. Subject labels are the same as Figure 2. Each subject is represented by a vertical line, with percent signal change to valid targets shown with circles, invalid targets with triangles, and oddball images with squares.

We also examined the robustness of vivid, novel oddball activation within the TPJ region defined by the spatial cueing validity contrast. As summarized in the “Introduction” section, a right hemisphere region in TPJ is the region most commonly identified in prior spatial cueing fMRI studies (see Table 1). In order to further examine the extent to which the oddball trials activate the same TPJ region as the attention reorienting trials, we defined a rh TPJ_STS_ ROI based on the group average contrast of invalid to valid trials. The group-defined ROI was mapped onto each individual subject in our set, resulting in an ROI with a mean surface area of 833 mm^2^. Although the ROI was defined using the “invalid vs. valid” contrast and was thus biased to see a robust effect of cue validity, oddball trials nonetheless drove the ROI more strongly than non-oddball trials (valid cue mean = 0.357 ± 0.134, valid cue followed by oddball mean = 0.696 ± 0.198, *t*_(9)_ = −5.1, *p* < 0.01; invalid cue mean = 0.490 ± 0.146, invalid cue followed by oddball mean = 0.715 ± 0.199, *t*_(9)_ = −3.4, *p* < 0.01; Figure 6). There was no rh TPJ_STS_ ROI in which the cue validity contrast yielded stronger activation than did the ViNO contrast. These results demonstrate that the vivid, novel oddball contrast is highly effective in identifying the rh TPJ region activated by the spatial cueing task.

### Comparison to Meta-Analysis

**Figure 7:**
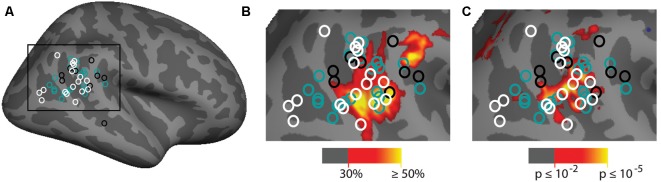
Meta-analysis of prior attention studies identifying TPJ compared with current group average task activation. **(A)** Meta-analysis of 45 prior studies using either spatial cueing (white), oddball tasks (black), or another attentional method (teal) to localize the right TPJ. **(B,C)** Zoom-in of meta-analysis overlaid on **(B)** probabilistic labels built from individual subject responses to the vivid, novel oddball contrast (see Supplementary Figure S5 and Supporting Information for further details) and **(C)** spatial cueing group-average activation maps. See Table 1 for a list of studies depicted.

The group analyses largely appear to replicate prior observations of attention-related activation in the vicinity of the TPJ. In order to put our findings in context, we also compared them to a meta-analysis of prior examinations of the TPJ region (see above). Two main contrasts were used in prior studies: spatial cueing and oddball-target tasks. In Figure 7A we plot the Talairach coordinates of prior attention-related localizations of TPJ with the localization method color-coded. Note that all of these studies are group averages. We compare this meta-analysis with probabilistic ROIs created from the individual subject ROIs defined from the vivid, novel oddball contrast (oddball trials vs. non-oddballs trials) in Figure 7B. In Figure 7C; we compare the meta-analysis with the group-activation pattern for valid vs. invalid (Figure 7C) trials. There is considerable overlap between our group average and the group averages of prior studies. The meta-analysis clustering is more strongly centered over TPJ_STS_ than TPJ_SMG_, but some prior oddball studies do overlap with TPJ_SMG_ as activated in the ViNO contrast here.

## Discussion

Attentional reorienting functions have long been attributed to a region where the temporal and parietal cortices abut on the lateral surface of the brain (Kincade et al., 2005; Fox et al., 2006; Corbetta et al., 2008; Shulman et al., 2009; Doricchi et al., 2010; Corbetta and Shulman, 2011; de Haan et al., 2015); however, the precise location of the attentionally activated region of the TPJ has proven to be very challenging due to anatomical variability across individuals and the general functional heterogeneity of this region of cortex (Hasson et al., 2004; Van Essen, 2005; Mueller et al., 2013). A challenge in functionally parsing TPJ is that it is very difficult to robustly drive fMRI BOLD responses in the visual attentional TPJ in individual subjects; a primary goal of the present study was to develop and validate a paradigm that supports identification of visual attentional responsive regions of TPJ in individual subjects. Here, we demonstrate that the presentation of vivid, novel visual oddball stimuli recruits two spatially distinct regions in the TPJ, a posterior region (TPJ_STS_) that spans both banks of the STS and an anterior region (TPJ_SMG_) that lies in the ventral portion of the SMG of the parietal lobe. Moreover, we demonstrate that these regions can be reliably identified in individual subjects. Since our task paradigm included both oddball events and spatial cueing components, we were able to validate that bilateral TPJ_STS_, identified in individual subjects with oddball images, also displays cue validity effects in a spatial reorienting task. These data demonstrate that right and left TPJ_STS_ are visually driven regions that are modulated by exogenous attention. Our findings also support a similar account for left TPJ_SMG_. In contrast, right TPJ_SMG_ did not conclusively exhibit modulation by exogenous attention and activation of this region in the ViNO paradigm may reflect only the visual drive or other factors unrelated to exogenous attention.

Our approach to developing an effective visual TPJ localizer builds on the findings of three prior studies in particular (Asplund et al., 2010; Horiguchi et al., 2016; Dugué et al., 2018). The use of visual drive to activate one or more visual TPJ regions has been shown to be effective in individual subjects (Horiguchi et al., 2016; Dugué et al., 2018). The reliance solely on the visual drive may be somewhat underpowered. Horiguchi et al. (2016) observed one visual TPJ in the right hemisphere of six of seven tested subjects but did not consistently observe visual drive within left hemisphere TPJ. However, Dugué et al. (2018) reliably observed multiple visual TPJ regions in each hemisphere of five subjects. Here, we sought to maximize visual drive by using highly vivid, full-field image stimuli. Horiguchi et al. (2016) additionally observed that visual oddball stimuli drove TPJ responses more strongly than did expected visual stimuli. Our approach combined the visual drive (sensory along with possible semantic and scene processing effects) and oddball factors (attention, surprise) by making each of the vivid images themselves the oddball stimuli. Our task paradigm was also inspired by the results of Asplund et al. (2010) who found that TPJ responses strongly attenuate with repeated oddball stimulus presentations; novelty is key. In order to maximize the response to each vivid oddball stimulus, we utilized a large set of oddball images and presented each stimulus only once to a subject. This vivid, novel oddball approach proved to be very effective in producing robust cortical responses in individual subjects. TPJ_STS_ was identified in 10/10 right hemispheres and 9/10 left hemispheres, while TPJ_SMG_ was identified in 6/10 right hemispheres and 5/10 left hemispheres. One caveat is that since eye movements were not monitored in these experiments, we cannot rule out possible contributions from eye movements. We suggest that because the oddball component of this task was task-irrelevant, the ViNO paradigm could be adapted for integration with other tasks. Task-irrelevant oddballs could be embedded in a wide variety of perceptual or cognitive tasks during fMRI scanning to localize TPJ regions in individual subjects while also collecting data in a primary task.

Corbetta and Shulman (2002); in delineating a Ventral Attention Network for processing of stimulus-driven attention, identified a node spanning the TPJ that ran from the superior temporal gyrus dorsally and anteriorly into the inferior parietal lobule. A number of more recent studies have suggested the existence of multiple functional subdivisions of TPJ (e.g., Igelström et al., 2015, 2016; Krall et al., 2015; Trautwein et al., 2016; Dugué et al., 2018); however, a consensus on nomenclature and functionality has yet to emerge. We identified two TPJ subdivisions serving attention and use anatomical landmarks to name them, TPJ_STS_ and TPJ_SMG_. While both TPJ_STS_ and TPJ_SMG_ were identified bilaterally (see also DiQuattro and Geng, 2011; de Haan et al., 2015), our results in Figure 2 are also consistent with prior reports that TPJ attentional reorienting is stronger in right hemisphere (Kincade et al., 2005; Fox et al., 2006; Corbetta et al., 2008; Shulman et al., 2009; Doricchi et al., 2010).

Although some prior studies examined more diverse cognitive phenomena, such as theory of mind, episodic memory retrieval, and default mode network effects, there are some noteworthy comparisons. Igelström et al. (2016) identified five TPJ subdivisions, with TPJ_c_ and TPJ_a_ exhibiting some functional similarities to TPJ_STS_ and TPJ_SMG_, respectively.

Dugué et al. (2018) also observed multiple visually responsive TPJ regions, two in the LH (vTPJ_ant_, vTPJ_post_) and three in the RH (vTPJ_ant_, vTPJ_cent_, vTPJ_post_). In contrast, Horiguchi et al. (2016) observed a single right-lateralized “visual TPJ” that responds to simple visual stimuli (drifting gratings, low-contrast dartboards) independent of context or expectation. Based on location and functional responses, our TPJ_SMG_ may correspond to vTPJ_ant_ (Dugué et al., 2018) and/or vTPJ (Horiguchi et al., 2016). Dugué et al. (2018) reported that vTPJ_ant_ was not modulated by exogenous nor endogenous attention in either hemisphere. Relatedly, we observed that the spatial cueing paradigm failed to attentionally modulate RH TPJ_SMG_; however, in an intriguing hemispheric asymmetry, LH TPJ_SMG_ was attentionally modulated. Dugué et al. (2018) reported visually-driven attentional modulations in two subregions (vTPJ_cent_, vTPJ_post_) lying on opposite banks of the STS. These regions appear similar to the present report of bilateral TPJ_STS_, but there are notable differences. Dugué et al. (2018) reported that vTPJ_post_ was observed bilaterally, while vTPJ_cent_ was observed in only one of five LHs. Although we also observed weaker TPJ_STS_ in LH than in RH, we observed no activation differences between banks of LH TPJ_STS_ in contrast to the Dugué et al. (2018) report. We observed distinct activation domains on each bank of STS in some individuals and single activation domains spanning both banks in other individuals. Our data do not resolve the question of whether these regions are functionally distinct from each other; however, we do not find evidence to support their reported hemispheric asymmetry for vTPJ_cent_, despite examining twice as many subjects. The discrepancies between the two sets of findings deserve further investigation; however, the similarities are notable.

The ability to localize TPJ regions in individual subjects should prove useful in more thoroughly characterizing the functionality of these regions. Attentional capacities vary across individuals (e.g., Fan et al., 2002) and these individual differences are relevant for Hemispatial Neglect Syndrome (e.g., Corbetta and Shulman, 2011; Rengachary et al., 2011) and other clinical patient groups (e.g., Bayliss and Tipper, 2010). The vivid, novel oddball contrast provides a novel approach to examine structure-function correlations. Moreover, the ability to identify attentional-driven regions of TPJ in individual subjects should be useful to address controversies over the precise functional organization of the TPJ. Notably, rh TPJ has been suggested to play a key role in temporal attention (Agosta et al., 2017). Another intriguing alternative hypothesis of TPJ function is the contextual updating model (DiQuattro and Geng, 2011; Geng and Vossel, 2013; DiQuattro et al., 2014), which relates to the P300 in the electroencephalography (EEG) literature (Donchin, 1981; Knight et al., 1989). The P300, or “oddball response,” is hypothesized to reflect updating of an internal model of the environment based on new sensory information or rare, unexpected events. Geng and Vossel (2013) propose that the computations performed by the TPJ are not right-lateralized or specific to attentional reorienting, but are instead reflective of contextual updating to an internal model of the environment. Although further research is required, the results described here are consistent with a contextual updating function of TPJ.

In summary, we demonstrate that the use of novel and vivid visual oddball stimuli can reliably reveal two bilateral visual attention regions in TPJ of individual subjects. Importantly, one region, the RH TPJ_STS_ corresponds to the region activated in a Posner-style spatial cueing task. The vivid, novel oddball contrast is task-irrelevant and could be integrated with a broad range of other tasks. We suggest that careful within-subject mapping across multiple functional tasks (e.g., attention, theory of mind) could prove useful for yielding a clearer understanding of the functional organization across the broad extent of the TPJ.

## Ethics Statement

All research participants were recruited from Boston University and the greater Boston community and gave written informed consent. All experiments were approved by the Institutional Review Board of Boston University. Participants were compensated monetarily for their time.

## Author Contributions

KD and DS designed the study and drafted the article. KD, MR and EL collected the data. KD and EL analyzed the data.

## Conflict of Interest

The authors declare that the research was conducted in the absence of any commercial or financial relationships that could be construed as a potential conflict of interest.
