# Supplementary material for: Identification of Visual Attentional Regions of the Temporoparietal Junction in Individual Subjects using a Vivid, Novel Oddball Paradigm
Source: Front Hum Neurosci. 2019 Dec 11;13:424. doi: 10.3389/fnhum.2019.00424 (PMC6917576; doi:10.3389/fnhum.2019.00424)
Supplement: Supplementary file 1 [file Data_Sheet_1.PDF]

Supporting Information:

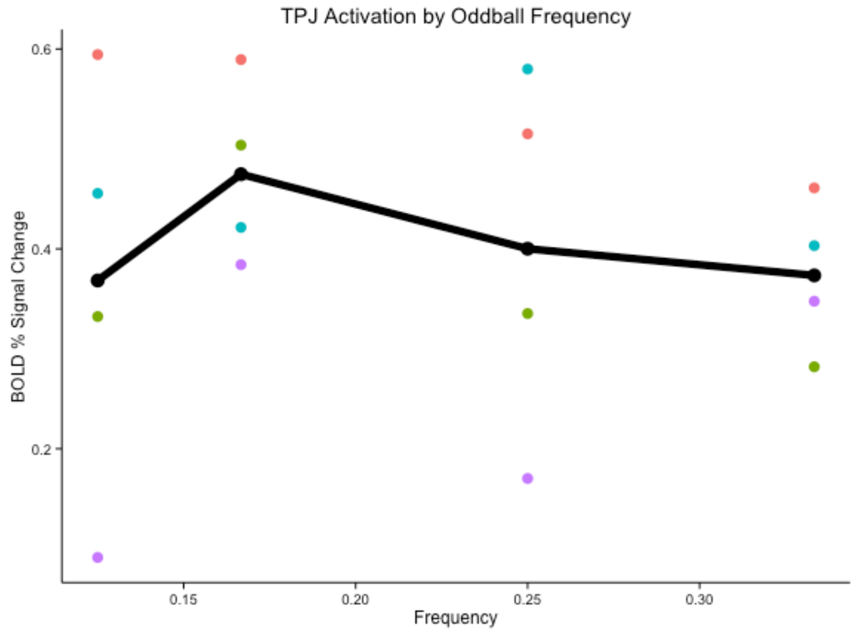

Supplementary Figure 1. Effects of Oddball Trial Frequency on BOLD activation in right TPJ_{STS}. In order to determine the optimal oddball distractor stimulus frequency, we ran a version of the task with the frequency of oddball presentations permuted across runs. Participants underwent 12 runs, with oddballs presented on either 1/4, 1/3, 1/6 and 1/8 of total trials per run, counterbalanced and randomized within participants. The right TPJ_{STS} region of interest (ROI) was defined individually in each subject using all oddball distractor trials compared to non-oddball trials ($p \leq 0.01$, uncorrected) over all 12 runs. We then conducted ROI-based analysis for each oddball frequency. Variation in task-evoked activity in TPJ showed a trend of an inverted U-shaped curve. Since, the 1/6 frequency (oddballs distractors on 16.67% of trials) yielded robust activation for all 4 subjects, this oddball frequency was used in the subsequent experiment. Individual subjects are shown as colored circles.

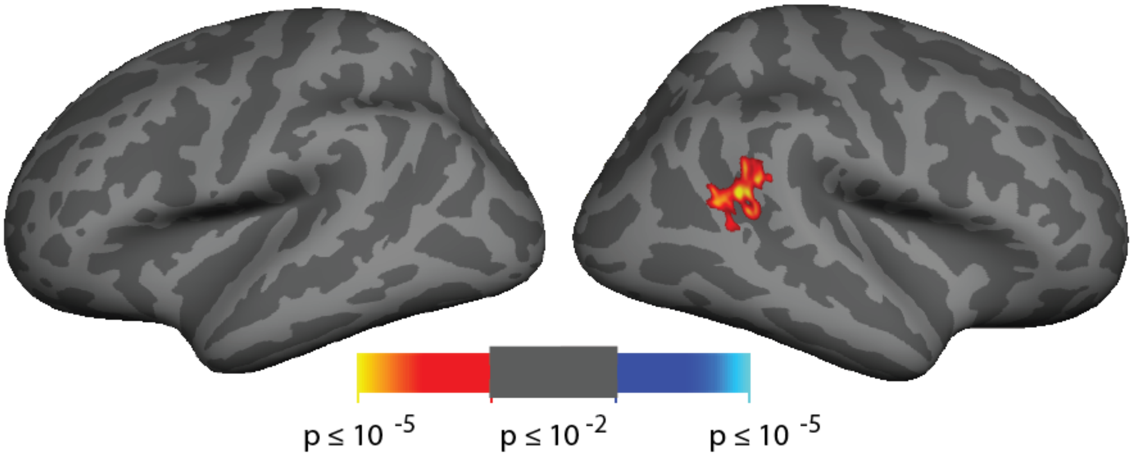

A)

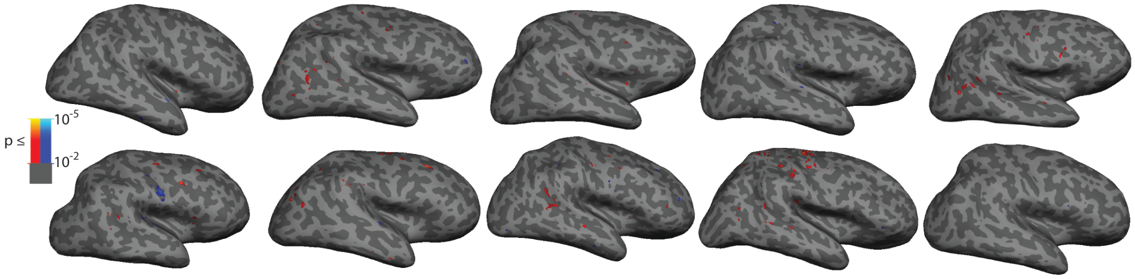

B)

Supplementary Figure 2 Additional Analysis of Spatial Cueing Contrast. A) Cluster Corrected Invalid vs. Valid Group Average. A group average analysis (N=10) of invalid vs. validly cued targets. Invalid targets activate the same right lateralized pTPJ as the oddball stimuli, but with lesser magnitude and spatial extent. Performing cluster correction eliminated all lateral invalid vs. valid activation in the LH. B) Individual subject RH activation in the contrast of invalid vs. valid cueing, thresholded at $p < 0.01$, uncorrected.

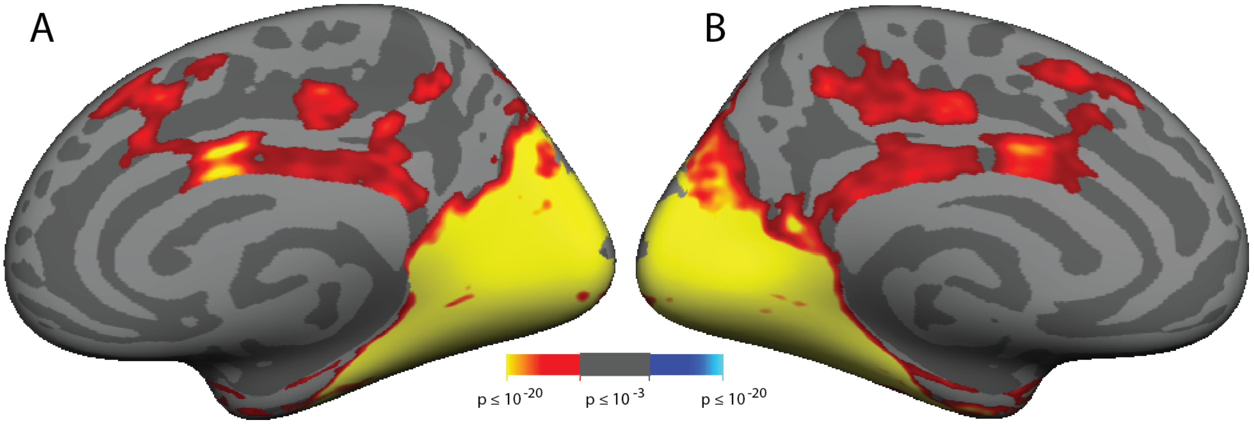

Supplementary Figure 3. Cluster corrected group average analysis (N=10) of oddball vs. non-oddball trials shown on the medial surface of the right (A) and left (B) hemispheres.

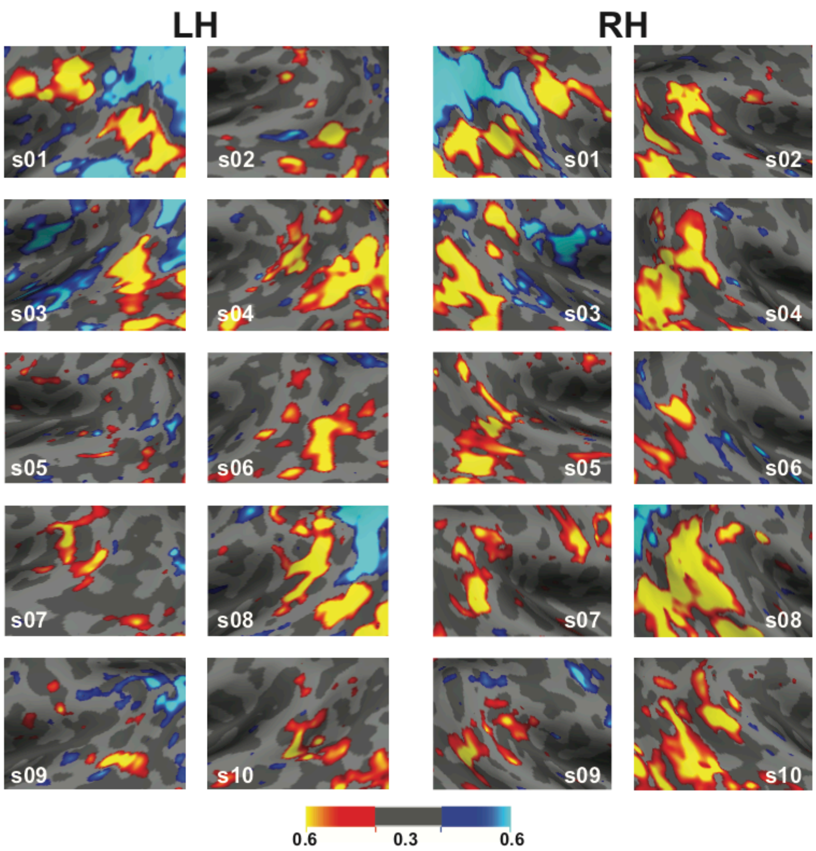

Supplementary Figure 4. Oddball vs. Non-Oddball Contrast Effect Size maps (ces.nii created by selxavg3-sess of FS-FAST) for each individual subject. Note the close similarity between the effect size maps shown here and the p-val maps shown in Figure 3B.

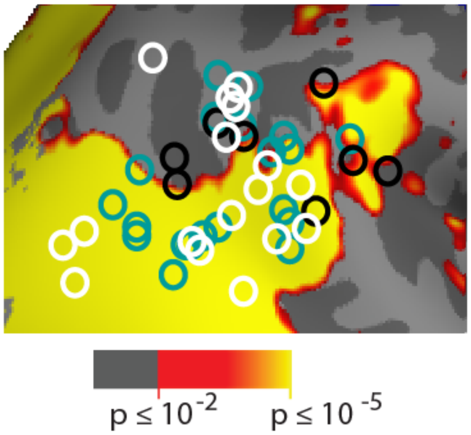

Supplementary Figure 5. Meta-analysis overlaid on group activation map for the contrast of Oddball > Non-Oddball trials. Statistical range shown to match that of the spatial cueing contrast shown in Figure 7C.

Oddball Probabilistic Labels were generated from each subjects individually defined region of interest shown in Figure 2B (rh TPJ_{STS}, n = 10; rh TPJ_{SMG}, n=6), based on the contrast of ‘Oddball Distractor vs. Standard Mask.’ Each region of interest was saved as a label (collection of vertices), transformed to the fsaverage brain and then averaged using the ‘mris_spherical_average’ tool. Each average label was subsequently thresholded at 0.3, meaning that a vertex must be active in at least 30% of individual subjects in order to be included in the label, and saved for visualization purposes.
